# Electrocardiographic abnormalities in children with idiopathic scoliosis

**DOI:** 10.1186/1748-7161-7-S1-O21

**Published:** 2012-01-27

**Authors:** J Durmala, M Sosnowska, M Sosnowski

**Affiliations:** 1 School of Health Care Katowice, Poland; 2 School of Medicine, Medical University of Silesia, Katowice, Poland

## Background

Cardiac involvement in the natural history of Idiopathic Scoliosis (IS) is not uncommon. We aimed at assessment of ECG abnormalities in a relatively large population of children with IS.

## Materials and methods

303 children, hospitalized in our Department, were examined. There were 260 girls and 43 boys, aged 14.2±0.2 and 14.1±0.4 years. Children with a certain diagnosis of cardiovascular disease were excluded. All patients had clinical and radiological evaluation. A routine ECG was recorded during in-hospital stay and analyzed for the presence of abnormal values/patterns in respect to age, gender and scoliosis gravity.

## Results

Any ECG abnormalities were found in 166 children (55%). In most of them 1 abnormal value/feature was seen (118, 39%), in 42 at least 2 (14%), and in 6 children >2 ECG pathologies were found (2%). The rsr’ pattern was most frequent (128, 42%). RVH was seen in 7 children (2%), LVH in 12 (4%) and LAE in 4 (1%). In 11 children (4%) abnormal HR was found, prolonged PR in 12 (4%), prolonged QRS in 23 (8%) and prolonged QTc in 1 (0.3%). P-wave axis deviation (11, 4%), QRS axis deviation (7, 2%), T-wave axis deviation (2, 1%) and abnormal ventricular gradient (9,3%) were also noticed. Presence of the ECG abnormalities did not depend on age, gender and the number or gravity of curvatures.

## Conclusions

There are number of abnormal ECG parameters in children with IS. Most of them are benign or negligible. More serious ECG abnormalities is uncommon, however, their presence should be more deeply evaluated.
